# Chrysin mitigates ciprofloxacin-induced nephrotoxicity: associations with notch/HES1 signaling, inflammation, and a ferroptosis-related molecular signature

**DOI:** 10.1007/s00210-026-05338-1

**Published:** 2026-04-15

**Authors:** Mustafa Önder Gönen, Nurhan Akaras, Hasan Şimşek, Özge Kandemir, Cüneyt Çağlayan, Serpil Aygörmez, Hüseyin Mutlu, Fatih Mehmet Kandemir

**Affiliations:** 1 Department of Emergency Medicine, Meram State Hospital, Konya, Türkiye; 2 Department of Histology and Embryology, Faculty of Medicine, Aksaray University, Aksaray, Türkiye; 3 Department of Physiology, Faculty of Medicine, Aksaray University, Aksaray, Türkiye; 4 Department of Food Processing, Vocational School of Technical Sciences, Aksaray University, Aksaray, Türkiye; 5 Department of Medical Biochemistry, Faculty of Medicine, Bilecik Seyh Edebali University, Bilecik, Turkey; 6 Department of Biochemistry, Faculty of Veterinary Medicine, Kafkas University, Kars, Türkiye; 7 Department of Emergency Medicine, Faculty of Medicine, Aksaray University, Aksaray, Türkiye; 8 Department of Medical Biochemistry, Faculty of Medicine, Aksaray University, Aksaray, Türkiye

**Keywords:** Ciprofloxacin, Chrysin, Nephrotoxicity, Ferroptosis, Notch signaling, IL-17A

## Abstract

Ciprofloxacin (CIP) is a widely used antibiotic frequently associated with dose-limiting nephrotoxicity. Chrysin (CHR), a natural flavonoid, possesses significant cytoprotective properties, yet its specific role in mitigating CIP-induced kidney injury remains underexplored. This study investigates the comprehensive nephroprotective mechanisms of CHR against CIP toxicity in rats. Male *Wistar* rats received CIP (100 mg/kg/day, i.p.) with or without CHR (50 mg/kg/day, p.o.) for 8 consecutive days. Results showed that CIP administration compromised renal function (urea, creatinine) and structural integrity, while markedly altering tubular injury markers (KIM-1, AQP-1). Biochemical and molecular analyses revealed that CIP disrupted cellular redox balance (MDA, GSH, SOD, CAT, GPx) and triggered a robust inflammatory response (NF-κB, TNF-α, IL-17A). Furthermore, CIP exposure engaged distinct cell death mechanisms, including intrinsic apoptosis (Bax, Bcl-2, Caspase-3) and ferroptosis (GPX4, TfR1, PTGS2). A novel finding was the activation of the Notch signaling pathway (Notch, HES1) in renal tissue. CHR co-treatment significantly attenuated these pathological changes, restoring renal function and histology, re-establishing antioxidant defenses, and suppressing inflammatory and apoptotic signaling and normalizing ferroptosis-related gene expression changes. In conclusion, CHR confers robust nephroprotection by targeting the inflammation-ferroptosis-apoptosis axis and modulating Notch signaling, positioning it as a promising adjuvant to mitigate CIP-induced renal injury.

## Introduction

Ciprofloxacin (CIP) is a broad-spectrum, second-generation fluoroquinolone antibiotic (Gauba and Saxena 2023). Due to its high oral bioavailability, extensive tissue penetration, and potent efficacy against a wide range of Gram-negative and Gram-positive pathogens, it is one of the most widely prescribed antibiotics globally for treating urinary, respiratory, gastrointestinal, and systemic infections (Millanao et al. 2021). Despite its significant therapeutic benefits, the clinical use of CIP is associated with several adverse effects, among which nephrotoxicity is a serious, dose-dependent complication (Badawy et al. 2021). This renal toxicity can manifest in various forms, including acute kidney injury (AKI), acute tubulointerstitial nephritis, and crystalluria (Morales-Alvarez 2020). The kidney is a primary target for CIP toxicity, largely due to the drug's tendency to accumulate within renal tubular epithelial cells, leading to high intracellular concentrations (Igbayilola et al. 2020). The pathophysiological mechanisms underlying CIP-induced nephrotoxicity are multifactorial, but oxidative stress is widely recognized as a central driver (Badawy et al. 2021). The metabolism of CIP is known to trigger the overproduction of reactive oxygen species (ROS), which overwhelms and depletes the cell's endogenous antioxidant defense systems, such as reduced glutathione (GSH), superoxide dismutase (SOD), catalase (CAT), and glutathione peroxidase (GPx). This severe redox imbalance results in extensive lipid peroxidation, as evidenced by increased malondialdehyde (MDA) levels, which in turn causes mitochondrial dysfunction and compromises cell membrane integrity (Delghandi et al. 2023). This initial oxidative damage serves as a primary trigger for a cascade of downstream dysregulated pathways (Caglayan et al. 2025). These include the activation of potent inflammatory responses, characterized by the upregulation of transcription factors like nuclear factor kappa B (NF-κB) and the release of pro-inflammatory cytokines such as tumor necrosis factor-alpha (TNF-α) and interleukin-17A (IL-17A) (Akhter et al. 2023; Rex et al. 2023). Concurrently, oxidative stress initiates programmed cell death pathways, primarily intrinsic apoptosis, which is marked by a shift in the B-cell lymphoma 2 (Bcl-2)-associated X protein (Bax)/Bcl-2 ratio and the activation of effector Caspase-3 (Hussar 2022). Together, these processes drive the loss of tubular epithelial cells and contribute to the progressive decline in renal function (Sanz et al. 2023). In addition to classical apoptosis, recent evidence suggests that ferroptosis-a distinct iron-dependent form of regulated cell death characterized by lipid peroxidation and glutathione peroxidase 4 (GPX4) inactivation-may contribute to drug-induced nephrotoxicity (Li et al. 2024). Unlike apoptosis, ferroptosis is driven by the accumulation of lipid-based reactive oxygen species and dysregulated iron metabolism, mediated by transferrin receptor 1 (TfR1) and prostaglandin-endoperoxide synthase 2 (PTGS2/COX-2) (Tang and Kroemer 2020). However, whether ferroptosis plays a role in CIP-induced renal injury has not been investigated. Beyond cell death pathways, developmental signaling cascades such as the Notch pathway may also be reactivated in response to renal injury (Wang et al. 2025). The Notch signaling pathway, particularly through its downstream transcriptional effector hairy and enhancer of split-1 (HES1), is a critical regulator of cell fate determination during kidney development. In the adult kidney, however, aberrant reactivation of Notch/HES1 has been implicated in maladaptive repair responses, including sustained inflammation, epithelial dedifferentiation, and progression to tubulointerstitial fibrosis (Luo et al. 2021). Notably, oxidative stress and pro-inflammatory cytokines-both hallmarks of CIP toxicity-are known to trigger Notch pathway activation in renal epithelial cells. Despite this, whether Notch/HES1 signaling contributes to CIP-induced nephrotoxicity remains unknown. Given the clinical importance of CIP, there is a pressing need for safe and effective adjunctive strategies that can mitigate its renal toxicity without compromising its antibacterial efficacy (Mohany et al. 2021).

Natural phytochemicals have emerged as highly promising candidates due to their pleiotropic (multi-target) properties (Bilginoğlu et al. 2025; Kızıl et al. 2025). Chrysin (CHR; 5,7-dihydroxyflavone) is a natural bioflavonoid found abundantly in honey, propolis, and various *Passiflora* species (Mohammed et al. 2023). A large body of literature has documented CHR's robust pharmacological activities, including potent antioxidant, anti-inflammatory, anti-apoptotic, and anti-fibrotic effects (Mani and Natesan 2018). Previous studies have successfully demonstrated its nephroprotective capacity against toxic insults from other agents, such as cisplatin and gentamicin (Albukhari et al. 2025; Shokrzadeh et al. 2025).

Recent studies suggest that pro-inflammatory cytokines, such as IL-17A, may create a permissive environment for ferroptosis by promoting lipid peroxidation, while aberrant Notch signaling often acts as an upstream regulator of this inflammatory maladaptation (Tang and Kroemer 2020; Chen et al. 2023; Song et al. 2023). To date, while the protective effects of CHR against other nephrotoxins are established, its specific efficacy and molecular mechanisms against CIP-induced nephrotoxicity remain unexplored. Hence, this study was designed to investigate whether CHR exerts renoprotective effects against CIP-induced nephrotoxicity in rats. We focused on the modulation of oxidative stress, inflammatory signaling (IL-17A/NF-κB), apoptosis (Bax/Bcl-2/Caspase-3), ferroptosis-related markers (PTGS2/GPX4/TfR1), and the Notch/HES1 pathway, and examined how these changes relate to structural and functional indices of renal injury.

## Materials and methods

### Reagents and chemicals

CIP and CHR were purchased from Sigma-Aldrich (St. Louis, MO, USA). All other chemicals and solvents used for biochemical and molecular analyses were of analytical grade and obtained from standard commercial suppliers.

### Animals and ethical approval

This study was conducted using twenty-eight (28) *Wistar albino* rats, aged 10–12 weeks and weighing between 220–250 g. The animals were obtained from and housed at the Necmettin Erbakan University Experimental Medicine Application and Research Center (KONÜDAM). The rats were kept in standard cages under controlled environmental conditions, including a constant temperature of 24–25 °C and a 12-h light/dark cycle (lights on 07:00–19:00). They were provided with standard pellet chow and tap water ad libitum. A one-week acclimatization period was allowed before the start of the experiments. All procedures were conducted in accordance with national and institutional guidelines for the care and use of laboratory animals (Approval Date: June 26, 2025, No: 2025–056).

### Experimental design and treatment protocol

Following the acclimatization period, the rats were randomly allocated into four groups (n = 7 per group). The specific dosage and administration protocol, based on the approved ethics application, were as follows:

- Control: rats received equivalent volumes of physiological saline orally and intraperitoneally once daily for 8 consecutive days.
- CHR: rats received CHR (50 mg/kg/day, p.o.) once daily for 8 consecutive days.
- CIP: rats received CIP (100 mg/kg/day, i.p.) once daily for 8 consecutive days.
- CIP + CHR: rats received CIP (100 mg/kg/day, i.p.) together with CHR (50 mg/kg/day, p.o.) once daily for 8 consecutive days.

The selected doses and duration were based on previous studies employing CIP‑induced nephrotoxicity and CHR nephroprotection models (Shaki et al. 2016; Kucukler et al. 2021), and were sufficient to induce reproducible renal injury without excessive mortality.

### Sample collection

At 24 h after the last treatment, rats were anesthetized with sevoflurane (3.5% for induction, 1.5–2% for maintenance). Blood was collected from the jugular vein into non-heparinized vacuum tubes, centrifuged at 3000 rpm for 10 min at 4 °C to obtain serum, and stored at −20 °C for biochemical analysis. Animals were then sacrificed by decapitation, and kidneys were rapidly excised. One kidney was allocated for biochemical assays and stored at −20 °C, whereas tissue samples intended for RT-PCR analyses were snap-frozen and stored at −80 °C until RNA extraction. The contralateral kidney was fixed in 10% neutral buffered formalin for histopathological examination.

### Biochemical analyses

#### Assessment of renal function

Serum was isolated by centrifuging blood samples at 1,500xg for 10 min at 4 °C. To assess renal function, urea and creatinine concentrations were determined using commercial kits from Diasis Diagnostic Systems (Istanbul, Türkiye).

#### Oxidative stress and antioxidant markers

Renal tissues were homogenized in ice-cold 1.15% KCl solution. The homogenates were centrifuged at 1000 × g for 15 min at 4 °C, and the supernatants were collected. Lipid peroxidation was assessed by measuring MDA levels spectrophotometrically at 532 nm using the thiobarbituric acid method described by Placer et al. (1966). The antioxidant status was evaluated by determining the levels of superoxide dismutase (SOD), catalase (CAT), and glutathione peroxidase (GPx) using rat-specific commercial ELISA kits (SunRed Biotechnology Company, Shanghai, China; Cat. No: 201–11–0169 for SOD, 201–11–5106 for CAT, and 201–11–1705 for GPx) according to the manufacturer's instructions. Reduced GSH levels were quantified according to the method of Sedlak and Lindsay (1968).

### Real-time PCR analysis

Total RNA was isolated from kidney tissues using the QIAzol-based extraction method (79,306; Qiagen, Germany) according to the manufacturer’s instructions, including chloroform phase separation, isopropanol precipitation, ethanol washing, and resuspension in DEPC-treated water. RNA concentration and purity were assessed spectrophotometrically at 260/280 nm using a NanoDrop system (Thermo Multiskan Go); all samples exhibited A260/A280 ratios between 1.8 and 2.0. Complementary DNA (cDNA) was synthesized from total RNA (2 µL) using the iScript cDNA Synthesis Kit (1,708,891; Bio-Rad, USA). Quantitative real-time PCR reactions were performed in a total volume of 20 µL containing 2 µL cDNA, gene-specific primers, and iTaq Universal SYBR Green Supermix (1,725,121; Bio-Rad, USA). Amplification was carried out using a Rotor-Gene Q system (Qiagen). The thermal cycling conditions consisted of an initial activation at 95 °C for 30 s, followed by 40 cycles of denaturation at 95 °C for 5 s and annealing/extension at 60 °C for 30 s. Melt-curve analysis (65–95 °C) was performed to confirm amplification specificity and absence of primer-dimers. The genes analyzed included inflammatory markers (NF-κB, TNF-α, IL-17A), apoptotic markers (Caspase-3, Bax, Bcl-2), ferroptosis-related genes (PTGS2, GPX4, TfR1), and components of the Notch signaling pathway (Notch, HES1). β-actin was used as the housekeeping gene for normalization, and relative gene expression levels were calculated using the 2^−ΔΔCt^ method (Livak and Schmittgen 2001). Standard curves were used to verify that primer efficiencies fell within the acceptable range (90–110%). Primer sequences and amplicon sizes for all target genes and β-actin are provided in Table 1. To ensure the reliability of the normalization, the stability of the housekeeping gene (β-actin) was validated by comparing the Cycle Threshold (Ct) values across all groups; no significant differences were observed (*p* > 0.05).

**Table 1:** Primer sequences

Gene	Sequences (5’−3’)	Length (bp)	Accession No
NF‑κB	F: AGTCCCGCCCCTTCTAAAAC R: CAATGGCCTCTGTGTAGCCC	106	NM_001276711.1
TNF‑α	F: CTCGAGTGACAAGCCCGTAG R: ATCTGCTGGTACCACCAGTT	139	NM_012675.3
Caspase‑3	F: ACTGGAATGTCAGCTCGCAA R: GCAGTAGTCGCCTCTGAAGA	270	NM_012922.2
Bax	F: TTTCATCCAGGATCGAGCAG R: AATCATCCTCTGCAGCTCCA	154	NM_017059.2
Bcl‑2	F: GACTTTGCAGAGATGTCCAG R: TCAGGTACTCAGTCATCCAC	214	NM_016993.2
PTGS2	F: CTCAGCCATGCAGCAAATCC R: GGGTGGGCTTCAGCAGTAAT	173	NM_017232
GPX4	F: TCTGAGCCGCTTATTGAAGCC R: CACACGCAACCCCTGTACTT	177	NM_017165
TFR1	F: CCGGCCTATATGCTTGGGTA R: CAAGGGAGCACTCTGAAGCA	103	NM_022712.1
Notch1	F: GTGGGATGGACTGGACTGTG R: GCGCAGGAAGTGGAAGGAGTT	117	NM_00110572
HES1	F: CGCCGGGCAAGAATAAATGA R: ATGTCTGCCTTCTCCAGCTT	104	NM_024360
β-Actin	F: CAGCCTTCCTTCTTGGGTATG R: AGCTCAGTAACAGTCCGCCT	360	NM_031144.3

### Histopathological examination

#### Hematoxylin and eosin (H&E)

Formalin-fixed kidney tissues were dehydrated through graded alcohol series, cleared in xylene, and embedded in paraffin wax. Sections of 5 μm thickness were cut using a microtome and stained with Hematoxylin and Eosin (H&E). The slides were examined under an Olympus Cx43 binocular light microscope equipped with an EP50 digital camera (Olympus Inc., Tokyo, Japan). Histopathological damage was evaluated using a semi-quantitative scoring system (0–4 scale) based on tubular dilatation, inflammatory cell infiltration, hemorrhage, vacuolization, and glomerular atrophy. All histopathological evaluations were performed by an experienced pathologist who was blinded to the experimental groups. The scores were defined as: 0 = no damage, 1 =  < 25%, 2 = 26–50%, 3 = 51–74%, and 4 =  ≥ 75% damage. Six randomly selected fields were analyzed per sample.

#### Immunohistochemical (IHC) analysis

Paraffin Sects. (3 μm thick) were deparaffinized and rehydrated. Antigen retrieval was performed by heating sections in citrate buffer (pH 6.0). Endogenous peroxidase activity was blocked with 3% hydrogen peroxide for 10 min. To prevent non-specific binding, sections were incubated with a protein block solution. The sections were then incubated overnight at 4 °C with primary antibodies against TIM-1/KIM-1 (PA1632, Boster Bio) and Aquaporin-1 (SC-25287, Santa Cruz Biotechnology), diluted 1:100 in PBS. This was followed by incubation with a secondary antibody and Streptavidin–Biotin complex. Immunoreactivity was visualized using 3,3’-diaminobenzidine (DAB) chromogen, and sections were counterstained with Harris hematoxylin. Staining intensity was graded as 0 (none), 1 (minimal), 2 (moderate), or 3 (intense). Quantitative density analysis was performed on five randomly selected fields per group using ImageJ software (NIH, Bethesda, MD, USA). Negative Controls were processed in parallel by omitting the primary antibody to exclude non‑specific staining.

### Statistical analysis

All experimental data are expressed as mean ± standard deviation (SD). Statistical analysis was performed using one-way analysis of variance (ANOVA) followed by Tukey's post hoc test for multiple comparisons. Differences were considered statistically significant at *p* < 0.05. Data visualization and statistical computations were conducted using GraphPad Prism version 9.5.0 (GraphPad Software, San Diego, CA, USA).

## Results

### CHR ameliorates CIP-induced renal dysfunction

**Fig. 1:**
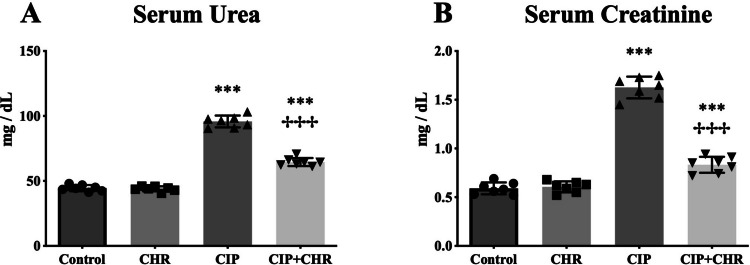
Effects of CIP and CHR on kidney function markers: (**A**) serum urea and (**B**) serum creatinine. Values are mean ± SD (*n* = 7/group). One-way ANOVA followed by Tukey’s post hoc test. *p < 0.05, **p < 0.01, ***p < 0.001 vs Control; + *p* < 0.05, +  + *p* < 0.01, +  +  + *p* < 0.001 vs CIP. Significance symbols are shown above the relevant groups

Administration of CIP resulted in a marked elevation in serum urea levels (95.78 ± 4.56 mg/dL), representing a 115.5% increase compared to the Control group (44.45 ± 2.38 mg/dL) (*p* < 0.001). Co-treatment with CHR (CIP + CHR group) significantly reduced urea levels to 64.53 ± 3.09 mg/dL, a 32.6% reduction relative to the CIP group (*p* < 0.001), indicating substantial functional recovery. Similarly, serum creatinine increased by 176.3% in the CIP group (1.63 ± 0.11 mg/dL) compared to Control (0.59 ± 0.06 mg/dL) (*p* < 0.001). CHR co-treatment significantly lowered creatinine levels to 0.83 ± 0.08 mg/dL, representing a 49.1% reduction versus CIP (*p* < 0.001) (Fig. 1).

### CHR restores antioxidant defenses and mitigates lipid peroxidation

**Fig. 2:**
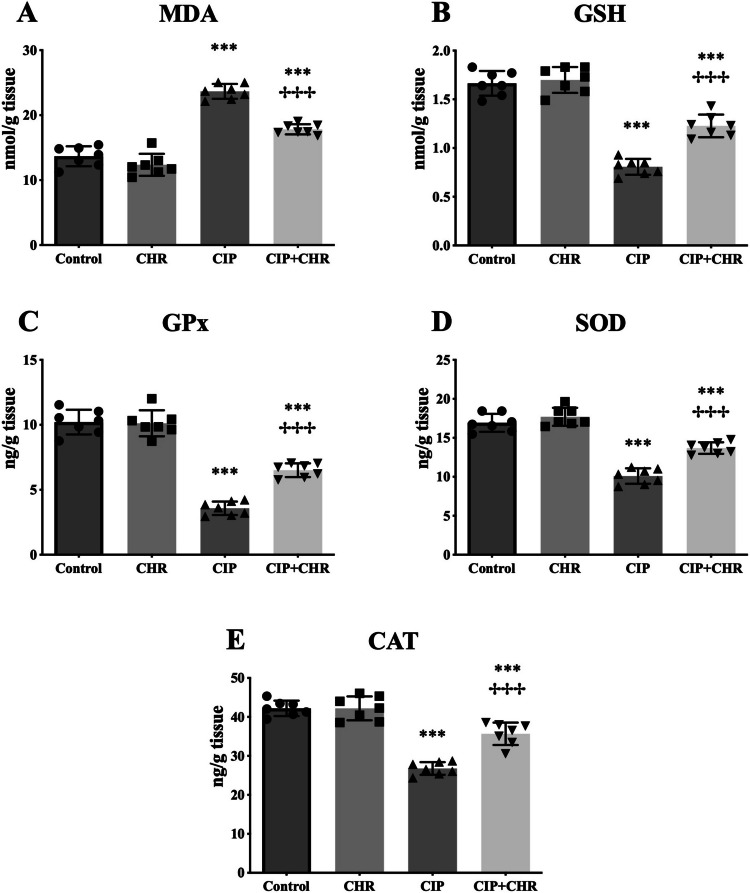
Effects of CIP and CHR on oxidative stress/antioxidant markers: (**A**) MDA, (**B**) GSH, (**C**) GPx, (**D**) SOD, and (**E**) CAT. Values are mean ± SD (*n* = 7/group). One-way ANOVA followed by Tukey’s post hoc test. *p < 0.05, **p < 0.01, ***p < 0.001 vs Control; + *p* < 0.05, +  + *p* < 0.01, +  +  + *p* < 0.001 vs CIP. Significance symbols are shown above the relevant groups

CIP administration triggered marked oxidative stress, increasing MDA levels by 73.1% (23.66 ± 1.15 nmol/g tissue) compared to Control (13.67 ± 1.54 nmol/g tissue) (*p* < 0.001). This elevation was significantly attenuated by 24.7% in the CIP + CHR group (17.82 ± 0.78 nmol/g tissue) (*p* < 0.001 vs CIP). Concurrently, CIP caused a 52.4% depletion of GSH (0.81 ± 0.08 nmol/g tissue) and significantly reduced enzymatic antioxidant levels: SOD by 40.3% (10.10 ± 0.99 ng/g tissue), CAT by 36.6% (26.77 ± 1.63 ng/g tissue), and GPx by 64.9% (3.58 ± 0.50 ng/g tissue) compared to Control (all *p* < 0.001). CHR co-treatment significantly reversed these deficits, increasing GSH by 55.7% (1.23 ± 0.08 nmol/g tissue), SOD by 35.5% (13.69 ± 0.51 ng/g tissue), CAT by 33.2% (35.67 ± 2.88 ng/g tissue), and GPx by 81.8% (6.51 ± 0.53 ng/g tissue) relative to the CIP group (all *p* < 0.001) (Fig. 2).

### CHR modulates gene expression of dysregulated pathways

#### Canonical inflammatory Axis (NF-κB/TNF-α) and IL-17A pathway

**Fig. 3:**
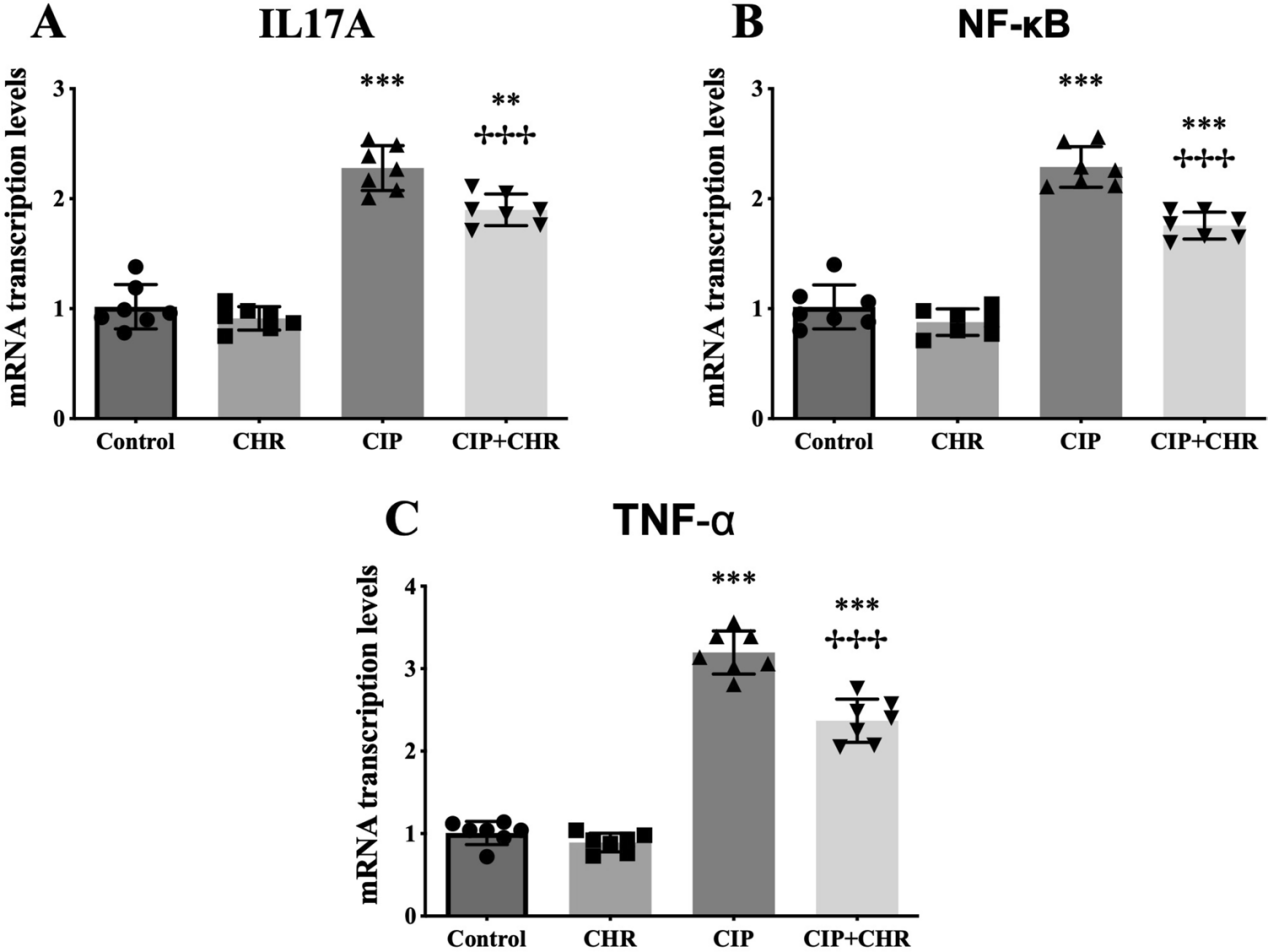
Effects of CIP and CHR on inflammatory gene expression: (**A**) IL-17A, (**B**) NF-κB, and (**C**) TNF-α. Relative mRNA expression was calculated by the 2^−ΔΔCt^ method, normalized to β-actin, and expressed relative to Control. Values are mean ± SD (*n* = 7/group). One-way ANOVA followed by Tukey’s post hoc test. *p < 0.05, **p < 0.01, ***p < 0.001 vs Control; + *p* < 0.05, +  + *p* < 0.01, +  +  + *p* < 0.001 vs CIP. Significance symbols are shown above the relevant groups

CIP administration induced a robust inflammatory response, characterized by a 2.25-fold increase in NF-κB expression (2.29 ± 0.18) compared to Control (1.02 ± 0.20) (*p* < 0.001). CHR co-treatment significantly downregulated NF-κB by 23.1% (1.76 ± 0.12) (*p* < 0.001 vs CIP). Similarly, TNF-α was upregulated by 3.17-fold (3.20 ± 0.26) in the CIP group and reduced by 25.9% with CHR treatment (2.37 ± 0.26) (*p* < 0.001 vs CIP). The IL-17A gene was upregulated by 2.24-fold (2.28 ± 0.20) due to CIP toxicity; however, CHR co-treatment significantly suppressed this expression by 16.7% to 1.90 ± 0.14 (*p* < 0.05 vs CIP) (Fig. 3).

#### Intrinsic apoptosis (Bax/Bcl-2/Caspase-3)

**Fig. 4:**
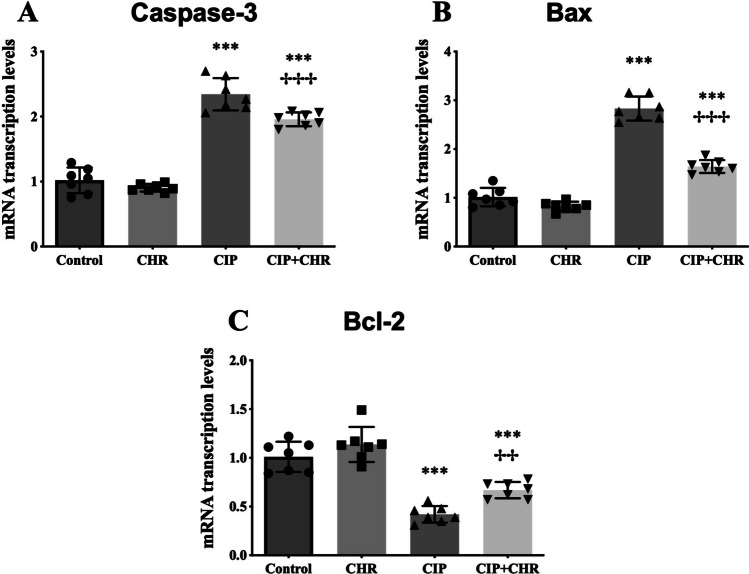
Effects of CIP and CHR on apoptotic gene expression: (**A**) Caspase-3, (**B**) Bax, and (**C**) Bcl-2. Relative mRNA expression was calculated by the 2^−ΔΔCt^ method, normalized to β-actin, and expressed relative to Control. Values are mean ± SD (*n* = 7/group). One-way ANOVA followed by Tukey’s post hoc test. *p < 0.05, **p < 0.01, ***p < 0.001 vs Control; + *p* < 0.05, +  + *p* < 0.01, +  +  + *p* < 0.001 vs CIP. Significance symbols are shown above the relevant groups

CIP treatment significantly upregulated the pro-apoptotic genes Caspase-3 (2.34 ± 0.25, 2.29-fold vs Control) and Bax (2.83 ± 0.25, 2.80-fold vs Control) while suppressing the anti-apoptotic gene Bcl-2 to 0.42 ± 0.08 (0.42-fold vs Control) compared to Control (*p* < 0.001). Co-administration of CHR effectively modulated these markers: it reduced Caspase-3 expression by 16.2% (1.96 ± 0.11) and Bax by 42.0% (1.64 ± 0.13), and significantly restored Bcl-2 levels by 59.5% (0.67 ± 0.08) compared to the CIP group (all *p* < 0.05) (Fig. 4).

#### Dysregulation of the GPX4/TfR1 axis and PTGS2 expression

**Fig. 5:**
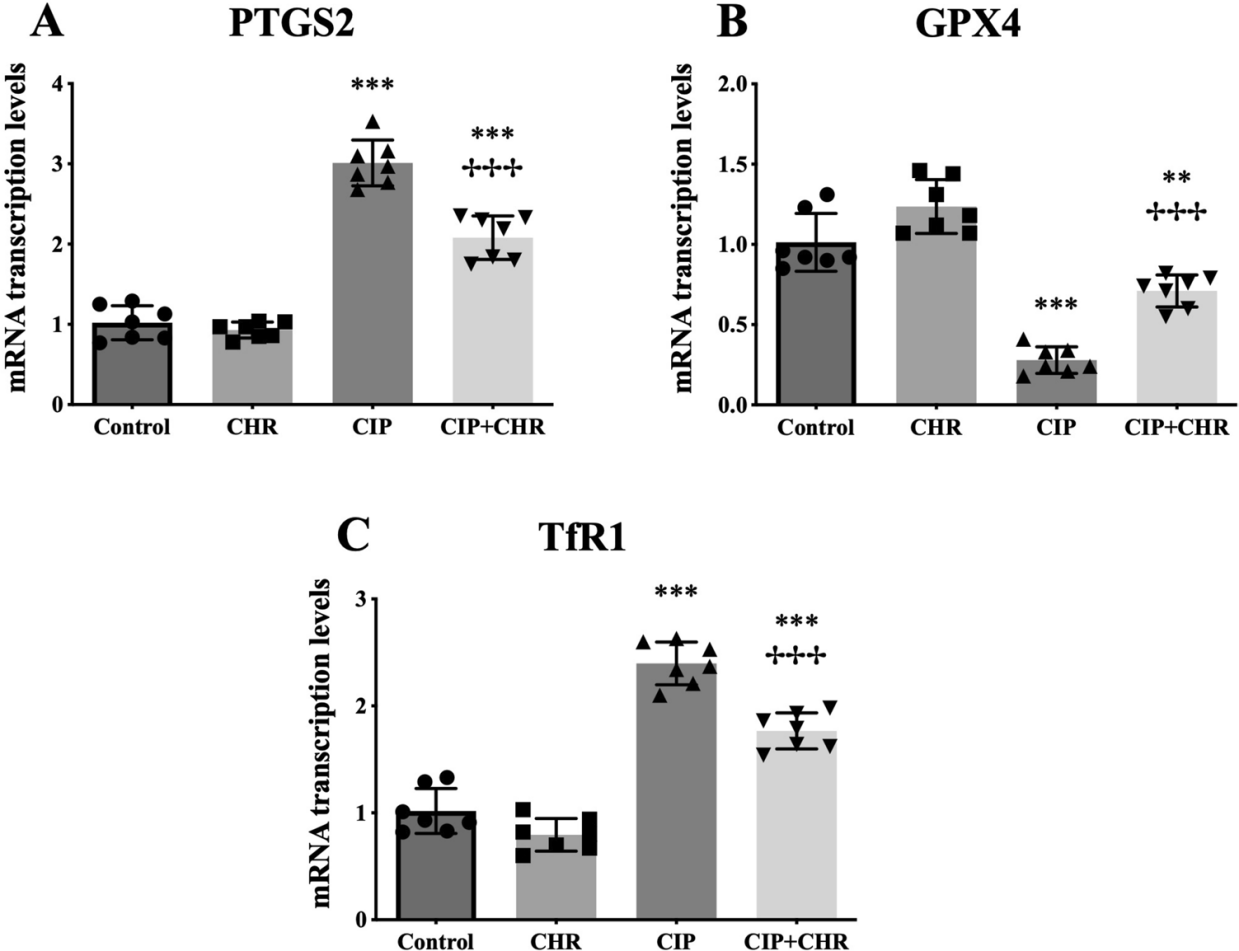
Effects of CIP and CHR on ferroptosis-related gene expression: (**A**) PTGS2, (**B**) GPX4, and (**C**) TfR1. Relative mRNA expression was calculated by the 2^−ΔΔCt^ method, normalized to β-actin, and expressed relative to Control. Values are mean ± SD (*n* = 7/group). One-way ANOVA followed by Tukey’s post hoc test. *p < 0.05, **p < 0.01, ***p < 0.001 vs Control; + *p* < 0.05, +  + *p* < 0.01, +  +  + *p* < 0.001 vs CIP. Significance symbols are shown above the relevant groups

In the CIP group, ferroptosis-related markers were significantly altered: PTGS2 was upregulated by 2.95-fold (3.01 ± 0.29), TfR1 by 2.35-fold (2.40 ± 0.20), while GPX4 was downregulated to 0.28 ± 0.08 (0.28-fold) relative to Control (1.01 ± 0.18) (*p* < 0.001). CHR co-treatment significantly mitigated this ferroptotic profile, reducing PTGS2 by 30.9% to 2.08 ± 0.27 and TfR1 by 26.2% to 1.77 ± 0.17, while dramatically increasing GPX4 expression by 153.6% to 0.71 ± 0.10 (all *p* < 0.001 vs CIP) (Fig. 5).

#### Notch signaling pathway (Notch/HES1)

**Fig. 6:**
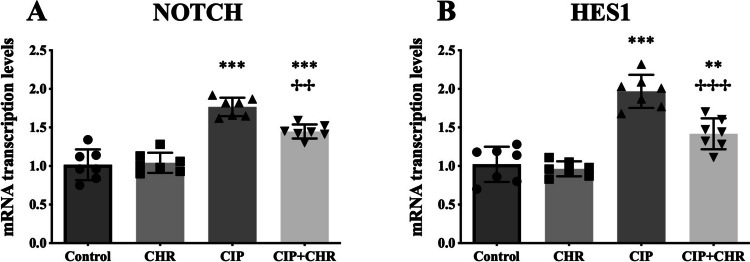
Effects of CIP and CHR on Notch pathway gene expression: (**A**) NOTCH and (**B**) HES1. Relative mRNA expression was calculated by the 2^−ΔΔCt^ method, normalized to β-actin, and expressed relative to Control. Values are mean ± SD (*n* = 7/group). One-way ANOVA followed by Tukey’s post hoc test. *p < 0.05, **p < 0.01, ***p < 0.001 vs Control; + *p* < 0.05, +  + *p* < 0.01, +  +  + *p* < 0.001 vs CIP. Significance symbols are shown above the relevant groups

CIP administration activated the Notch pathway, evidenced by a 1.74-fold increase in Notch expression (1.77 ± 0.12) and a 1.93-fold increase in HES1 (1.97 ± 0.22) mRNA levels (*p* < 0.001 vs Control: 1.02 ± 0.20 and 1.02 ± 0.23, respectively). CHR co-treatment significantly suppressed this activation, reducing Notch expression by 18.1% to 1.45 ± 0.09 and HES1 by 27.9% to 1.42 ± 0.20 (*p* < 0.05 vs CIP) (Fig. 6).

### Histological and immunohistochemical analysis

#### Histopathological analysis of renal architecture and damage scoring

**Fig. 7:**
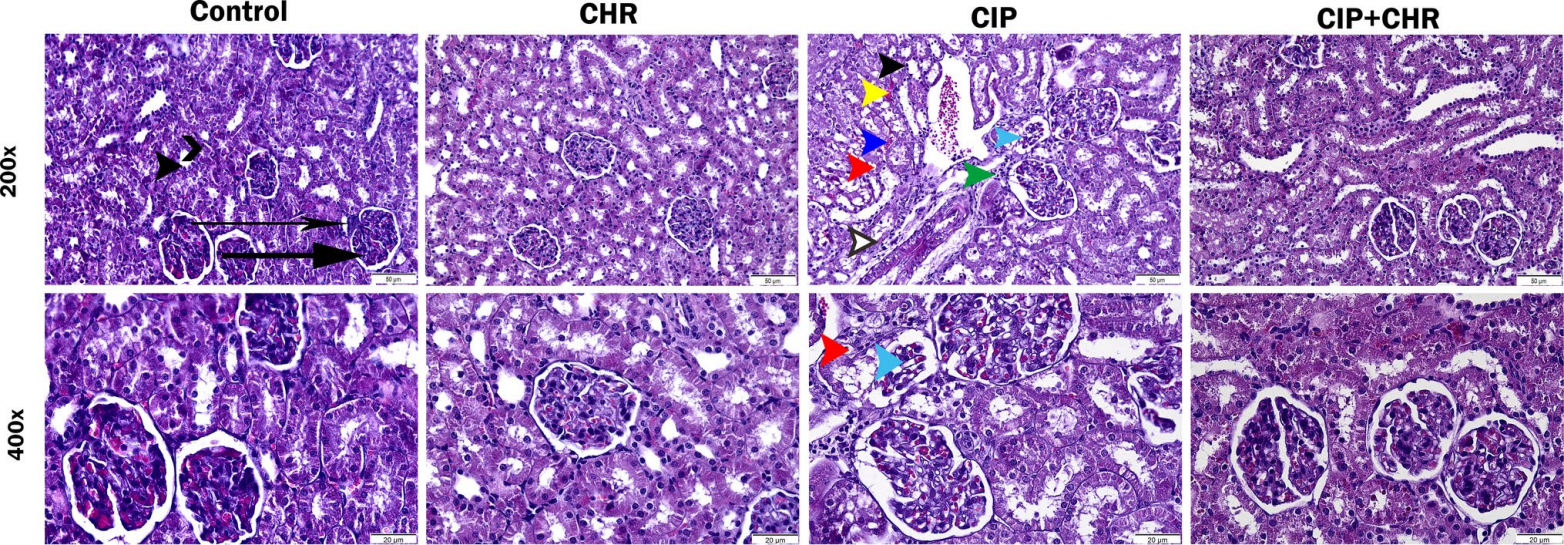
Microscopic images of kidney tissues stained with Hematoxylin & Eosin (H&E) from different experimental groups (Scale bar = 50, 20 µm) were evaluated. In the kidney tissues of the Control and CHR groups, renal corpuscles (thick arrow) and the macula densa (thin arrow) appeared with normal morphology; the Bowman’s space was of normal width, and the proximal (arrow tip) and distal (arrow head) tubular epithelial cells maintained their integrity. In the CIP group, structural deformities were observed in the glomeruli (light blue arrowhead), pyknotic nuclei in tubular epithelial cells (black arrowhead), cytoplasmic vacuolization (red arrowhead), and epithelial cell desquamation in certain areas (white arrowhead). Additionally, interstitial edema (yellow arrowhead), mononuclear cell infiltration (green arrowhead), vascular congestion, and focal hemorrhage (blue arrowhead) were noted. In the CIP + CHR-treated group, histopathological damage was markedly reduced; glomerular structural integrity was largely preserved, tubular degeneration was alleviated, and no significant disruption was observed in interstitial vascular structures

Microscopic examination of H&E-stained kidney sections demonstrated that renal histological architecture was well-preserved in both the Control and CHR-only treated animals. These groups exhibited regular glomerular morphology with a standard Bowman's space width, alongside intact epithelial lining of the proximal and distal tubules (Fig. 7). No signs of degeneration, necrosis, or interstitial alterations were detected, confirming the renal safety of CHR administration alone.

Conversely, CIP administration precipitated severe renal parenchymal injury. The CIP-treated group was characterized by marked glomerular deformation and significant expansion of the Bowman's space. Tubular toxicity was evident, manifesting as widespread cytoplasmic vacuolization, the presence of pyknotic nuclei, and the desquamation of epithelial cells into the tubular lumen. The interstitial compartment displayed distinct pathological features, including edema, extensive mononuclear cell infiltration, vascular congestion, and areas of focal hemorrhage. Furthermore, tissue shrinkage was observed, leading to the widening of extracellular spaces between tubules and vascular structures.

Co-administration of CHR (CIP + CHR group) remarkably attenuated these histopathological aberrations. The integrity of glomerular and tubular structures was largely maintained, with only minimal inflammatory cell infiltration and preserved vascular architecture observed in the interstitial space. Semi-quantitative scoring (Table 2) corroborated these observations; CIP treatment resulted in statistically significant elevations in scores for tubular dilatation (3.28 ± 0.48), inflammatory cell infiltration (3.00 ± 0.57), hemorrhage (3.57 ± 0.53), vacuolization (3.14 ± 0.37), and glomerular atrophy (3.42 ± 0.78) compared to Controls (*p* < 0.05). CHR significantly mitigated these damage indices (e.g., tubular dilatation: 1.42 ± 0.53; hemorrhage: 1.57 ± 0.53), confirming its structural protective efficacy (*p* < 0.05).

**Table 2:** Histopathological and immunohistochemical findings and scores in kidney tissue in rats

Parameters	Control	CHR	CIP	CIP + CHR
Tubular dilatation	0,28 ± 0,48^+^	0,14 ± 0,37^+^	3,28 ± 0,48*	1,42 ± 0,53*^+^
Inflammatory cell infiltration	0,14 ± 0,37^+^	0,14 ± 0,37^+^	3,00 ± 0,57*	1,14 ± 0,37*^+^
Hemorrhage	0,42 ± 0,53^+^	0,42 ± 0,53^+^	3,57 ± 0,53*	1,57 ± 0,53*^+^
Vacuolization	0,42 ± 0,53^+^	0,28 ± 0,48^+^	3,14 ± 0,37*	1,42 ± 0,53*^+^
Glomerular atrophy	0,57 ± 0,53^+^	0,42 ± 0,53^+^	3,42 ± 0,78*	1,57 ± 0,53*^+^
Aquaporin-1 immun expression	2,71 ± 0,48^+^	2,85 ± 0,37^+^	0,28 ± 0,48*	2,00 ± 0,57*^+^
KIM/TIM-1 immun expression	0,28 ± 0,48^+^	0,28 ± 0,48^+^	2,85 ± 0,37*	1,28 ± 0,48*^+^

Data are mean ± SD. *(*p* < 0.05) compared to Control groups, ^+^(*p* < 0.05) compared to CIP group

#### Immunohistochemical analysis of renal injury

**Fig. 8:**
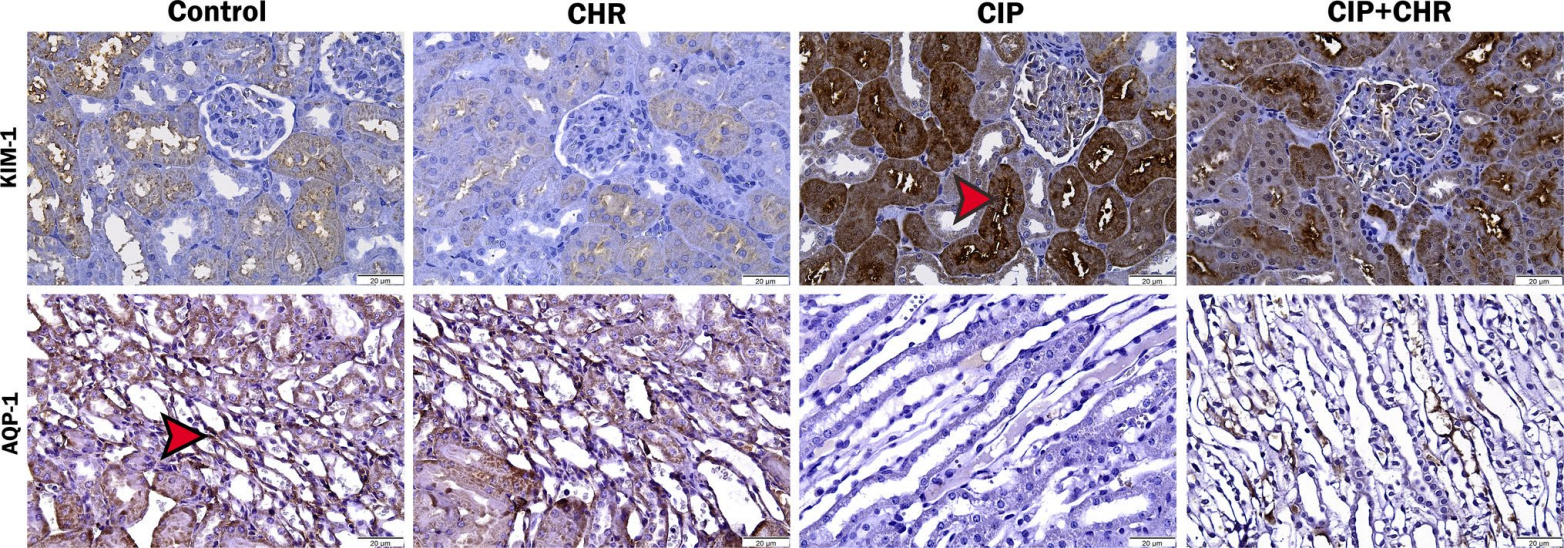
Immunohistochemical expression of KIM-1 and AQP-1 in kidney tissues from different experimental groups. KIM-1 expression was minimal or nearly negative in the Control and CHR groups, whereas in the CIP-treated groups, intense cytoplasmic positivity was observed in the proximal tubular epithelium (arrowhead). AQP-1 staining showed strong cytoplasmic positivity (arrowhead) in the Control and CHR groups, while this positivity was significantly reduced in the CIP-treated groups. In the CIP+CHR-treated group, partial recovery of AQP-1 expression and a significant decrease in KIM-1 levels were observed. IHC, DAB chromogen, hematoxylin counterstaining; scale bar = 20 µm

Immunohistochemical analysis was performed to evaluate the specific expression patterns of KIM-1 and Aquaporin-1 (AQP-1) as markers of tubular injury and membrane integrity, respectively (Fig. 8, Table 2). As a sensitive biomarker of proximal tubule injury, KIM-1 immunoreactivity was negligible in tissues from Control and CHR-treated rats, with scores of 0.28 ± 0.48. In sharp contrast, CIP exposure triggered a dramatic upregulation of KIM-1, visualized as intense cytoplasmic positivity specifically localized to the proximal tubular epithelial cells (Score: 2.85 ± 0.37). CHR co-treatment significantly suppressed this upregulation, reducing both the intensity and extent of KIM-1 staining (Score: 1.28 ± 0.48; *p* < 0.05), thereby indicating a substantial reduction in proximal tubular damage.

Regarding functional integrity, AQP-1 expression was strongly and diffusely observed in the proximal tubules, loop of Henle, and vasa recta of Control (2.71 ± 0.48) and CHR-treated (2.85 ± 0.37) groups. CIP administration resulted in a profound loss of AQP-1 immunoreactivity, particularly within the loop of Henle, where staining was weak, irregular, or absent (Score: 0.28 ± 0.48). CHR intervention significantly restored AQP-1 expression patterns (2.00 ± 0.57; *p* < 0.05), suggesting the effective preservation of tubular membrane stability and water transport channels against CIP-induced toxicity.

## Discussion

CIP remains a cornerstone in the management of severe bacterial infections; however, its clinical application is severely constrained by the incidence of acute kidney injury. While the pathophysiology of this nephrotoxicity is multifactorial, oxidative stress and the subsequent activation of aberrant inflammatory and cell death signaling are established as central drivers (Shah et al. 2022). CHR, a bioactive flavonoid, possesses potent cytoprotective properties (Mohammed et al. 2023), yet its specific molecular efficacy in the context of CIP-induced renal damage has remained underexplored. The present study provides robust biochemical and molecular evidence that CHR confers nephroprotection against CIP toxicity by orchestrating a multi-targeted defense strategy: it restores redox homeostasis, suppresses the Notch/HES1 signaling axis, and mitigates the pathological interplay involving IL-17A-driven inflammation, apoptosis, and ferroptosis.

Oxidative stress is widely recognized as the prime mover in the pathogenesis of fluoroquinolone-induced nephrotoxicity (Fuller 2015). Mitochondrial dysfunction and the consequent surge in ROS overwhelm endogenous antioxidant defenses, triggering the dysregulated cascade (Gencer et al. 2024). Biochemical indices such as MDA, a marker of lipid peroxidation, and the enzymatic triad of SOD, CAT, and GPx, alongside reduced GSH, characterize this redox imbalance (Akaras et al. 2023; Kankılıç et al. 2024). The present study, CIP administration precipitated a marked oxidative insult, demonstrated by elevated MDA levels and a markedly impaired antioxidant defense system. This redox imbalance is not merely a bystander but a key early driver that compromises cellular membrane integrity. CHR treatment effectively reversed this state, functioning not merely as a radical scavenger but as a strong inducer of enzymatic defenses. By restoring the cellular redox balance at the beginning of the injury cascade, CHR likely limits the mitochondrial perturbations that fuel downstream damage pathways.

**Fig. 9:**
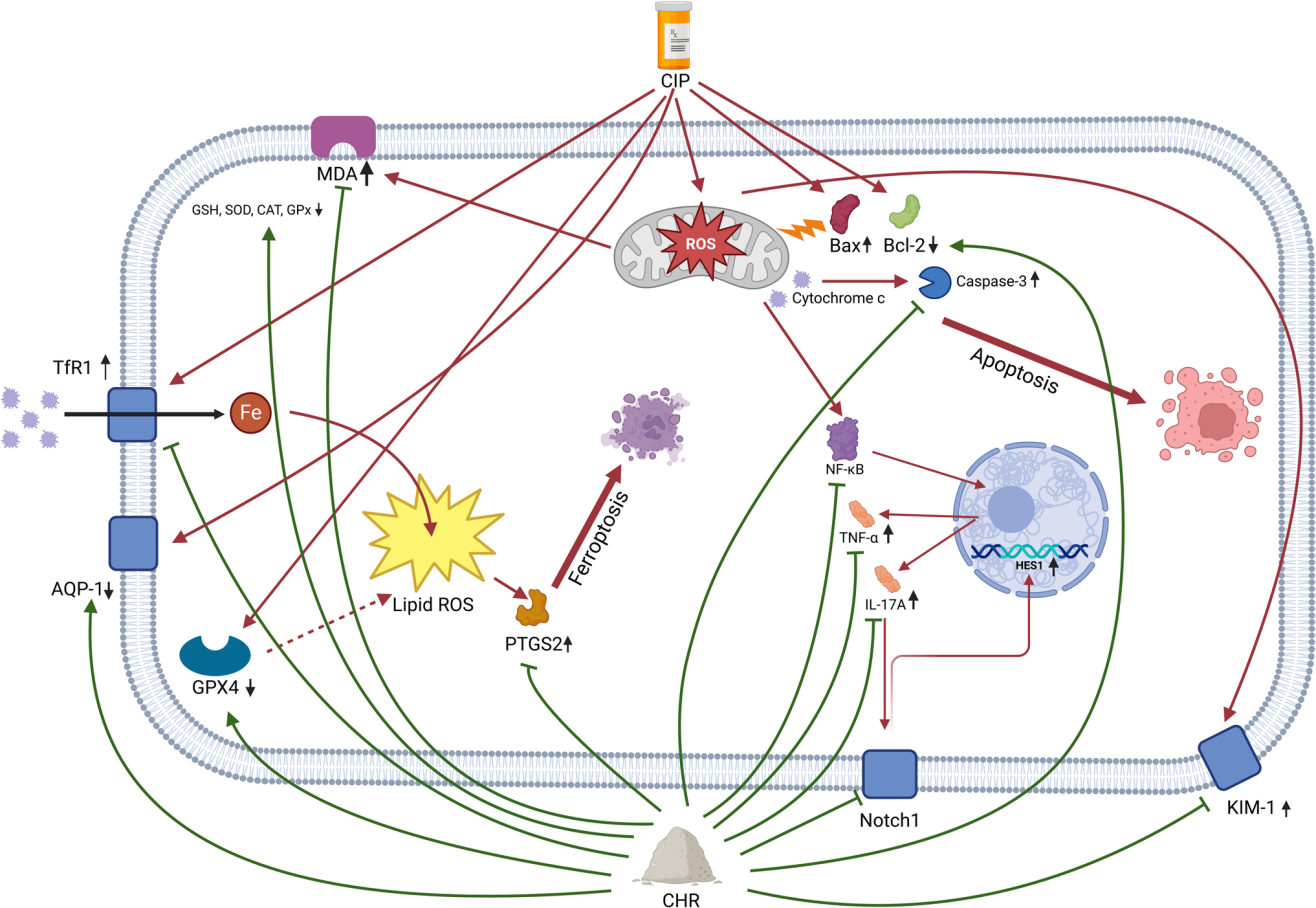
Schematic representation of the proposed nephroprotective mechanism of chrysin (CHR) against ciprofloxacin (CIP)-induced nephrotoxicity. CIP exposure is associated with oxidative stress, reflected in this study by increased lipid peroxidation (↑MDA) and depletion of antioxidant defenses (↓GSH, SOD, CAT, GPx), and with dysregulation of inflammatory signaling (↑IL-17A/NF-κB/TNF-α), Notch/HES1 activation, and a ferroptosis-related transcript signature (↓GPX4, ↑TfR1, ↑PTGS2). These pathways crosstalk and converge to promote tubular injury and renal dysfunction. CHR mitigates CIP-induced kidney injury by improving the antioxidant/oxidative stress profile (↓MDA; partial restoration of GSH, SOD, CAT, GPx, and GPX4 toward control levels), suppressing inflammatory and Notch/HES1 signaling, and normalizing ferroptosis-related markers (T-bars indicate inhibition; arrows indicate stimulation/induction). Created in BioRender. Kizil, H. (2025) https://BioRender.com/to0k9wz

Following the initial oxidative insult, the activation of inflammatory signaling propagates and amplifies tissue injury (Kızıl 2025). NF-κB acts as a central transcriptional regulator of this response, driving the expression of downstream effectors such as TNF-α and IL-17A, which sustain the inflammatory microenvironment (Darendelioglu et al. 2025; Zhu et al. 2025). A decisive downstream consequence of the oxidative stress in the present model was the activation of this self-perpetuating inflammatory loop. Crucially, our study highlights the involvement of IL-17A, a potent pro-inflammatory cytokine known to recruit neutrophils and exacerbate tissue destruction (Mills 2023). The upregulation of IL-17A in CIP nephrotoxicity indicates a critical immune-mediated component to the injury. CHR significantly attenuated this inflammatory response by downregulating IL-17A, NF-κB, and TNF-α. This finding suggests that the protective mechanism of CHR involves the disruption of the immune-inflammatory crosstalk, thereby limiting secondary tissue injury that occurs after the initial toxic hit. Importantly, IL-17A has been shown to crosstalk with both Notch and ferroptotic pathways (Fig. 9). IL-17A can directly activate Notch signaling in epithelial cells, amplifying inflammatory responses and promoting fibrotic remodeling (Wang et al. 2021). Additionally, IL-17A-driven inflammation enhances lipid peroxidation and iron accumulation, creating a permissive environment for ferroptosis (Song et al. 2023). Thus, the suppression of IL-17A by CHR may represent a key upstream intervention that simultaneously disrupts multiple dysregulated axes-inflammation, Notch activation, and ferroptosis-thereby explaining the pleiotropic protective effects observed in the present study.

Simultaneously, developmental signaling pathways such as Notch are reactivated, potentially acting as integrators of these stress signals (Shi et al. 2024). The Notch/HES1 axis is an evolutionarily conserved pathway that regulates cell fate; however, its persistent reactivation in the adult kidney is increasingly linked to maladaptive repair, inflammation, and tubulointerstitial fibrosis (Luo et al. 2021). An important finding of this study is the identification of the Notch signaling pathway as a potential therapeutic target in CIP nephrotoxicity. We found that CIP exposure significantly upregulated both the Notch receptor and its downstream effector HES1. This activation likely reinforces the inflammatory milieu and contributes to tubular cell dysfunction. Importantly, CHR co-treatment significantly suppressed the Notch/HES1 pathway. This downregulation suggests that CHR exerts its broad protective effects-anti-inflammatory, anti-apoptotic, and attenuating ferroptosis-associated molecular alterations at least in part through the upstream modulation of Notch signaling, thereby attenuating the transcriptional progression of renal injury. The mechanism by which CHR suppresses Notch/HES1 activation is likely indirect. Oxidative stress and pro-inflammatory cytokines, particularly TNF-α and IL-17A, are known to activate Notch signaling in renal epithelial cells (Marquez‐Exposito et al. 2021; Lin et al. 2022). By restoring redox balance and suppressing NF-κB-driven inflammation, CHR may prevent the upstream signals that trigger Notch pathway reactivation. Alternatively, flavonoids have been shown to directly interfere with Notch receptor processing and γ-secretase activity (Mondal et al. 2021), though this mechanism requires further investigation in the context of CIP nephrotoxicity.

Programmed cell death pathways are major determinants of tubular cell loss in toxic nephropathies, with intrinsic apoptosis emerging as a critical mechanism (Aygörmez et al. 2025). Intrinsic apoptosis is governed by the delicate balance between pro-apoptotic proteins, such as Bax, and anti-apoptotic guardians like Bcl-2 (Kızıl et al. 2023). Under conditions of severe cellular stress, Bax translocates to the mitochondria, inducing outer membrane permeabilization and the release of cytochrome c, which in turn activates the executioner Caspase-3 (Emre Kızıl et al. 2023). In the present study, CIP administration significantly disrupted this balance, upregulating Bax and Caspase-3 while suppressing Bcl-2. This shift confirms the activation of the intrinsic apoptotic cascade, likely triggered by the upstream mitochondrial oxidative stress we observed. CHR co-treatment successfully modulated these markers, downregulating Bax/Caspase-3 and restoring Bcl-2 expression. This finding aligns with previous reports demonstrating the anti-apoptotic efficacy of flavonoids in renal injury models and suggests that CHR preserves nephron mass by directly inhibiting the mitochondrial pathway of apoptosis (Genç et al. 2025).

The convergence of oxidative stress, sustained inflammation, and aberrant signaling ultimately dictates tubular cell fate through regulated cell death pathways, extending beyond apoptosis to ferroptosis (Zhou et al. 2022). The present study reveals that CIP toxicity associated with a distinct ferroptosis-related phenotype, characterized by the suppression of GPX4 (a key regulator of ferroptosis), iron overload via TfR1 upregulation, and enhanced lipid peroxidation reflected by PTGS2. The upregulation of TfR1 facilitates intracellular iron accumulation, which, in the presence of oxidative stress and reduced GPX4, catalyzes membrane damage (Xie et al. 2024). CHR co-treatment successfully rebalanced this axis by restoring GPX4 levels and reducing TfR1 and PTGS2 expression. Although these changes do not by themselves prove overt ferroptosis, they delineate a ferroptosis-related stress signature in CIP-treated kidneys and suggest that CHR preserves tubular cell viability by modulating ferroptosis-related molecular changes in addition to inhibiting apoptosis. The identification of a ferroptosis-related phenotype in CIP nephrotoxicity has potential therapeutic implications. Unlike apoptosis, ferroptosis can be pharmacologically targeted with iron chelators (e.g., deferoxamine) or lipophilic antioxidants (e.g., ferrostatin-1). Our findings suggest that combination strategies-pairing CHR with ferroptosis inhibitors-may offer synergistic renoprotection. Moreover, the dual engagement of apoptotic and ferroptotic pathways may explain why single-target interventions have shown limited efficacy in previous studies of fluoroquinolone toxicity.

The cumulative effect of these cellular injuries manifests as functional organ impairment and structural damage. Serum urea and creatinine, the primary biochemical surrogates for glomerular filtration (Gur et al. 2022; Gur and Kandemir 2023), were markedly elevated following CIP administration, consistent with significant impairment of renal function. This functional decline was substantiated histologically by widespread proximal tubular necrosis, glomerular atrophy, and the loss of brush border integrity (Çomaklı et al. 2022). Molecularly, this was confirmed by the dramatic downregulation of Aquaporin-1 (AQP-1), essential for constitutive water reabsorption, and the profound upregulation of KIM-1, a highly sensitive biomarker of proximal tubular injury (Lv et al. 2024). CHR co-treatment significantly restored AQP-1 expression, suppressed KIM-1 upregulation, improved renal function tests, and preserved histological architecture. These outcomes provide strong supporting evidence that CHR’s intervention at the molecular level-by dampening oxidative stress, inflammation, and Notch signaling-is associated with meaningful organ-level protection.

The present results establish a clear association between CHR treatment and the transcriptional suppression of the Notch/HES1 axis. While the current dataset does not resolve whether this signature reflects a direct interference with Notch receptor processing or, more plausibly, an indirect consequence of attenuated oxidative and inflammatory cues, the consistent downregulation of these markers supports the involvement of Notch/HES1 signaling within the broader renoprotective landscape of CHR. Defining the precise biochemical interface linking CHR to Notch pathway attenuation remains an important objective for future mechanistic work.

From a translational standpoint, although CHR conferred marked nephroprotection in this model, the possibility that it may modulate CIP disposition and/or antibacterial performance warrants consideration. In this context, flavonoids have been discussed as potential antibiotic adjuvants, partly through modulation of bacterial efflux systems, with in vitro evidence suggesting that CHR can potentiate CIP activity in resistant *E. coli* (Waditzer and Bucar 2021; Lu et al. 2025). Nonetheless, given that CHR may interact with cytochrome P450 dependent metabolism, targeted pharmacokinetic and PK/PD evaluations will be necessary to clarify the net consequence of co-administration and to ensure that toxicity mitigation is not achieved at the expense of bactericidal efficacy. One limitation of our study is that we relied primarily on transcriptional profiling without complementary protein-level confirmation of key signaling nodes. Additionally, we did not directly assess inflammatory cell infiltration using specific IHC markers. Therefore, while gene expression patterns are coherent, the precise cellular composition and spatial distribution of the inflammatory response cannot be fully delineated.

## Conclusion

In conclusion, the present study demonstrates that CHR emerges as a promising nephroprotective agent against CIP induced renal injury. Its mechanism of action is pleiotropic, involving the restoration of oxidative balance, the preservation of tubular water channels (AQP-1), and the inhibition of tubular injury markers (KIM-1). Mechanistically, CHR appears to act by suppressing dysregulated Notch/HES1 signaling and mitigating the pathological interplay involving IL-17A-driven inflammation, ferroptosis-related molecular alterations, and apoptosis. These findings position CHR as a candidate adjuvant to expand the therapeutic window of CIP and may help mitigate its dose limiting nephrotoxicity.

## Data Availability

The datasets generated and analyzed during the current study are fully presented within this published article and its accompanying figures and tables. The raw data supporting the findings of this study (including original qPCR CT values and specific statistical output files) are not publicly archived but are available from the corresponding author, Assoc. Prof. Dr. Hüseyin Mutlu, upon reasonable request.
